# A four-year observational study to examine the dietary impact of the North Carolina Healthy Food Small Retailer Program, 2017–2020

**DOI:** 10.1186/s12966-021-01109-8

**Published:** 2021-03-24

**Authors:** Stephanie B. Jilcott Pitts, Qiang Wu, Kimberly P. Truesdale, Ann P. Rafferty, Lindsey Haynes-Maslow, Kathryn A. Boys, Jared T. McGuirt, Sheila Fleischhacker, Nevin Johnson, Archana P. Kaur, Ronny A. Bell, Alice S. Ammerman, Melissa N. Laska

**Affiliations:** 1 Department of Public Health, Brody School of Medicine, East Carolina University, Greenville, NC 27834 USA; 2 Department of Biostatistics, East Carolina University, Greenville, NC 27834 USA; 3 Department of Nutrition, University of North Carolina at Chapel Hill, Chapel Hill, NC 27599 USA; 4 Department of Agricultural & Human Sciences, North Carolina State University, Raleigh, 27695 USA; 5 Department of Agricultural & Resource Economics, North Carolina State University, Raleigh, 27695 USA; 6 Department of Nutrition, University of North Carolina at Greensboro, Greensboro, 27412 NC USA; 7 Georgetown University Law Center, Washington, DC, 20001 USA; 8 Department of Social Sciences and Health Policy, Division of Public Health Sciences, Wake Forest School of Medicine, Winston-Salem, 27157 USA; 9 Wake Forest Baptist Comprehensive Cancer Center, Winston-Salem, NC 27157 USA; 10 Center for Health Promotion and Disease Prevention, University of North Carolina at Chapel Hill, Chapel Hill, NC 27599 USA; 11 Healthy Weight Research Center, University of Minnesota School of Public Health, Minneapolis, MN 55454 USA

**Keywords:** Healthy corner stores, Food environment, Health policy, Food desert, Rural, Fruits and vegetables

## Abstract

**Background:**

The North Carolina (NC) Healthy Food Small Retailer Program (HFSRP) was passed into law with a $250,000 appropriation (2016–2018) providing up to $25,000 in funding to small food stores for equipment to stock healthier foods and beverages. This paper describes an observational natural experiment documenting the impact of the HFSRP on store food environments, customers’ purchases and diets.

**Methods:**

Using store observations and intercept surveys from cross-sectional, convenience customer samples (1261 customers in 22 stores, 2017–2020; 499 customers in 7 HFSRP stores, and 762 customers in 15 Comparison stores), we examined differences between HFSRP and comparison stores regarding: (1) change in store-level availability, quality, and price of healthy foods/beverages; (2) change in healthfulness of observed food and beverage purchases (“bag checks”); and, (3) change in self-reported and objectively-measured (Veggie Meter®-assessed skin carotenoids) customer dietary behaviors. Differences (HFSRP vs. comparison stores) in store-level Healthy Food Supply (HFS) and Healthy Eating Index-2010 scores were assessed using repeated measure ANOVA. Intervention effects on diet were assessed using difference-in-difference models including propensity scores.

**Results:**

There were improvements in store-level supply of healthier foods/beverages within 1 year of program implementation (0 vs. 1–12 month HFS scores; *p* = 0.055) among HFSRP stores only. Comparing 2019 to 2017 (baseline), HFSRP stores’ HFS increased, but decreased in comparison stores (*p* = 0.031). Findings indicated a borderline significant effect of the intervention on self-reported fruit and vegetable intake (servings/day), though in the opposite direction expected, such that fruit and vegetable intake increased more among comparison store than HFSRP store customers (*p* = 0.05). There was no significant change in Veggie Meter®-assessed fruit and vegetable intake by customers shopping at the intervention versus comparison stores.

**Conclusions:**

Despite improvement in healthy food availability, there was a lack of apparent impact on dietary behaviors related to the HFSRP, which could be due to intervention dose or inadequate statistical power due to the serial cross-sectional study design. It may also be that individuals buy most of their food at larger stores; thus, small store interventions may have limited impact on overall eating patterns. Future healthy retail policies should consider how to increase intervention dose to include more product marketing, consumer messaging, and technical assistance for store owners.

## Background

Over the past decade, the number of public health nutrition interventions and policies related to healthy corner store initiatives has increased [1–3]. These initiatives have been promoted as strategies to improve diet-related behaviors and, ultimately, to reduce risk of diet-related diseases, particularly in underserved areas [1, 2]. Some of these initiatives have been the result of local or statewide policies and/or financial appropriations to improve the availability of healthy food and beverage items in small food store environments [4]. For example, the Minneapolis Staple Foods Ordinance in Minnesota was the first policy requiring food stores to stock minimum amounts and varieties of healthy foods and beverages through licensing [5]. Examining changes in food store environments, customer purchases, and home food environments annually pre- (2014) and post- (2015–17) implementation of this ordinance, Laska et al. found stores were compliant, but there were no statistically significant improvements in relevant outcomes, such as the healthfulness of customer purchases or the healthfulness of home food environments among frequent shoppers, when comparing stores in Minneapolis to those in the control city, St. Paul, Minnesota [5].

One commonly cited barrier to stocking and promoting healthier food options in small food stores is a lack of refrigeration and equipment needed to store and display perishable foods [6–8]. Prior evaluations of healthy corner store initiatives, or voluntary programmatic strategies aimed at increasing healthy food stocking via equipment, technical assistance, and other support have found improvements in the stocking of healthy foods and beverages [9–13]. Findings have been mixed, however, concerning the impact on customers’ purchase and consumption of healthy foods with some studies finding positive effects [10, 12, 14] and others finding no effects [5, 11] of these programs. Many prior healthy corner store evaluation studies, however, have had relatively small customer sample sizes (ranging from *n* = 84 to *n* = 401 [2, 3]), a limited number of post-intervention follow-up measures, used self-reported consumption data, and/or did not include a comparison group of stores [2, 3, 15]. Gittelsohn et al., noted the great need for, but challenges associated with, obtaining accurate and reliable customer dietary data to evaluate healthy small store initiatives [15].

Between 2016 and 2018, the North Carolina (NC) state legislature annually appropriated $250,000 to the Department of Agriculture and Consumer Services (NCDA&CS) to implement the NC Healthy Food Small Retailer Program (HFSRP). The HFSRP funds were distributed to small stores in US Department of Agriculture (USDA)-defined food deserts to purchase refrigeration equipment to stock healthier foods and beverages.

### Overview of the NC HFSRP

**Fig. 1:**
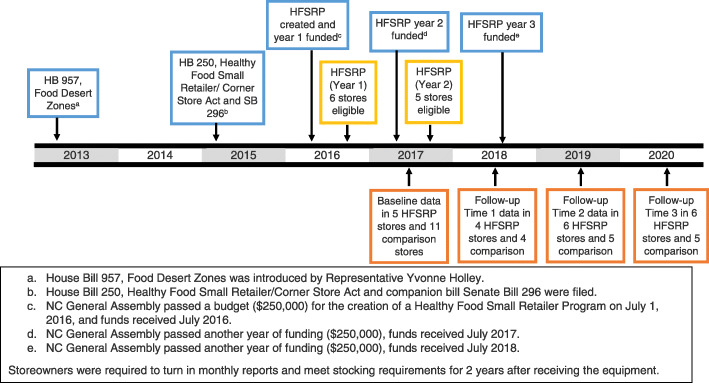
A timeline of the North Carolina Healthy Food Small Retailer Program and associated activities

Figure 1 provides a timeline of the HFSRP and associated activities. In 2013, House Bill 957, was introduced by Representative Yvonne Holley. This later became House Bill 250, the Healthy Food Small Retailer/Corner Store Act and companion bill, Senate Bill 296. The NC General Assembly passed a budget ($250,000) for the creation of a Healthy Food Small Retailer Program on July 1, 2016, and funds were received July 2016. Thus, in July 2016, 2017, and 2018, the HFSRP was funded through an appropriations bill, allocating $250,000 per year ($750,000 total) to be administered through the NCDA&CS to small food retailers [12]. The HFSRP was administered in the form of small (maximum of $25,000 per store) grants for refrigeration equipment to stock and promote healthier foods. Stores were eligible if they were located in USDA-defined food deserts, occupied 3000 heated square feet or less, and accept or agree to accept Supplemental Nutrition Assistance Program (SNAP) benefits and accept or agree to apply to accept Special Supplemental Nutrition Program for Women, Infants, and Children (WIC) benefits. Funding could only be used “for the purchase and installation of refrigeration equipment, display shelving, and other equipment necessary for stocking nutrient-dense foods, including fresh vegetables and fruits, whole grains, nuts, seeds, beans and legumes, low-fat dairy products, lean meats, and seafood.” [16] HFSRP stores were required to stock and promote healthy foods in the HFSRP equipment for at least 24 months [16]. Stores signed an agreement stating they would stock healthier foods and beverages for 24 months; after this period, store owners could stock this equipment with any items they wished. Based upon data collected by the NCDA&CS, [17] for the 2016–17 cohort of stores, the minimum amount funded was $16, 878.71 and maximum was $25,000. For the 2017–18 cohort, the minimum was $3984.32 and maximum was $16,003.96. The majority of the funding was used for coolers, refrigeration units and shelving. There were no official campaigns conducted to promote the new healthy foods and beverages but stores often worked with the local health departments on promoting the healthier options.

The purpose of this study is to report results of an observational study of stores participating in the first 3 years of the HFSRP, as well as matched comparison stores to document the impact of the HFSRP on store food environments, customer purchases, and customers’ diets between 2017 and 2020. More specifically, we examined the: (1) change in store-level availability, quality, and price of healthy foods and beverages in HFSRP and comparison stores; (2) change in diet quality of customer purchases of foods and beverages from HFSRP and comparison stores; and (3) change in self-reported and objectively measured customer dietary behaviors.

## Methods

### Selection of stores for the HFSRP and for the observational study of dietary impact

Selection of HFSRP stores was on a rolling basis between 2016 and 2019 through an application process [11, 17]. For our observational study, we collected data in all HFSRP stores that allowed it, with one store not allowing intercept surveys. Comparison stores were systematically selected, and were matched on factors including North American Industry Code Standards (NAICS) store type and store size (square footage) information from the ReferenceUSA business database, census tract USDA food desert designation, and census tract American Community Survey 2012–2016 5-year estimates demographic characteristics of the store’s local area (percent of the census tract using SNAP benefits, percent African American residents) (Table 1).

**Table 1:** Store type, year, and number of intercept surveys per year among seven Healthy Food Small Retailer Program (HFSRP) Stores and 15 Comparison stores, 2017–2020

Store type (HFSRP vs Comparison)	year				HFSRP (Yes/No)	
	2017	2018	2019	2020	No	Yes
HFSRP Store A	0	0	25	24	0	49
HFSRP Store B	31	29	0	0	0	60
HFSRP Store C	32	36	25	25	0	118
HFSRP Store D ^a^	0	0	22	0	0	22
HFSRP Store E	32	0	29	25	0	86
HFSRP Store F	37	30	29	25	0	121
HFSRP Store G	29	14	0	0	0	43
Comparison Store A ^b^	0	0	30	26	56	0
Comparison Store B	16	0	0	0	16	0
Comparison Store C	37	21	0	0	58	0
Comparison Store D	30	0	0	0	30	0
Comparison Store E	27	0	0	0	27	0
Comparison Store F	30	0	0	0	30	0
Comparison Store G	0	0	31	25	56	0
Comparison Store H	22	0	0	0	22	0
Comparison Store I	30	30	27	26	113	0
Comparison Store J	0	0	28	25	53	0
Comparison Store K	34	31	16	0	81	0
Comparison Store L	49	32	25	30	136	0
Comparison Store M	23	0	0	0	23	0
Comparison Store N	20	0	0	0	20	0
Comparison Store O	0	0	27	14	41	0

^a^This store had HFSRP equipment in it when baseline measures were collected, although the equipment was broken at the time

^b^This was an HFSRP store but did not have equipment when data were collected, so it was treated as a control

### Store-level healthy food supply (HFS) score

The HFS score was used to measure store-level availability, price, and quality of foods and beverages. We used the HFS score because it was currently being used by another large ongoing studies in small food retailers (thus our results could be comparable to other policy-relevant research findings with small food retailers), and it was a validated tool [18, 19]. We were also interested in assessing the impact of the policy on overall healthfulness of the store environment, such as whether retailers would make other healthy changes in the store as a result of program participation. The HFS Score is derived from the validated Nutrition Environment Measures-Stores audit tool, used in prior studies [18, 19].

The methods described by Andreyeva et al. [18] were used to assign HFS scores. Audits were conducted in each store, each year. Audit data included store characteristics, such as type of store (convenience/corner store, food/gas mart, dollar store, pharmacy, or other), SNAP authorization, number of cash registers and number of aisles. In addition, the audit included information on availability, quality, and price of fruits, vegetables, dairy products, protein sources, whole grains, and other food items [19]. The HFS score considers: availability of soy milk, tofu, and canned sardines and salmon; price and availability of cow’s milk; availability and varieties of brown rice, whole-grain bread, cereals, tortillas, canned and frozen fruit and vegetables; and availability, varieties, and quality of fresh fruit and vegetables. Scores range from 0 to 31 points with higher scores awarded to stores stocking healthier food and beverages. Using audit data, two or more study team members independently calculated HFS scores for each store. The study team then discussed the independently-derived HFS scores, and reconciled any scoring discrepancies.

### Store-level healthy eating index (HEI)-2010 of customer purchases

Store-level HEI scores were calculated based on foods and beverages purchased from customers during “bag checks,” as described previously [11]. For each item purchased, product name, brand, size, quantity and price paid were recorded. Among the customers who completed customer intercept surveys (*n* = 1261), 1224 completed a bag check (89.1%). The National Cancer Institute (NCI) Automated Self-Administered 24-h recall website (ASA24) was used to determine the overall nutrient profile of purchases at the store level. Items from all customers at each store were included and a single, aggregated, Healthy Eating Index (HEI)-2010 score was calculated for each store, which had a potential range from 0 to 100.

### Store-level sample size justification

Using 2017 baseline and 2018 follow-up data, [11] HFSRP stores had a mean pre-post difference in HFS score of + 3.1 while the control stores had a mean pre-post difference of − 0.4. Thus, group sample sizes of eight HFSRP and eight comparison stores would achieve 85% power to detect a population mean difference of 3.1 (conservative estimate) with a standard deviation for both groups of 1.94 (the standard deviation of our 2017–18 data) when a significance level (alpha) of 0.05 is used in a two-sided two-sample equal-variance t-test. In each year, we were limited by the number of stores who received HFSRP funding, the stores that were within a reasonable travel distance (one HFSRP store was too far from our study team for surveys to be conducted) and, among these, the number of stores that allowed us to conduct evaluation measures at their stores (one HFSRP store refused at the beginning, and one control store allowed evaluation measures for 2 years, but did not allow the measures midway through the second year).

### Individual-level customer intercept survey

We conducted customer intercept surveys in control and HFSRP stores, in February to May of each year (2017–2020). The goal was to survey 25–30 customers per store, based on feasibility given data collection resources and constraints; the number of customers surveyed per store ranged from 14 to 49, with a mean of 27.4 customers surveyed per store over the 4 years of data collection. A convenience sample of customers were asked to complete the questionnaire, bag check, and Veggie Meter® scans (described below) after their store purchases. We interviewed every customer willing to be interviewed while the research assistants were in the stores. Intercept surveys were conducted during normal business hours on weekdays. Customers were eligible to participate if they were over 18 years of age and spoke English. Participating customers provided verbal informed consent and were offered a $10 gift card to Wal-Mart as an incentive for participating. The study was approved by the East Carolina University Institutional Review Board (UMCIRB 16–002420).

### Individual-level, customer dietary outcomes

Using the approach described by Townsend et al., self-reported fruit and vegetable (FV) intake was measured using two single item questions, one for fruits and another for vegetables [20]. The fruit question was as follows: “On a typical day, how many servings of fruits do you eat? (A serving of fruit is like a medium sized apple or a half cup of fresh fruit—this does not include fruit juice)” with responses reported as whole numbers. The National Cancer Institute (NCI) FV Screener [21] was used as a second measure of FV intake. We also asked if participants had previously purchased FV at the store. While the HFSRP legislation did not explicitly address sugary beverages, we hypothesized that customers may substitute some of their sugary beverage choices with healthier beverage choices in HFSRP stores. To assess sugary beverage consumption, items from the Behavioral Risk Factor Surveillance System Survey were used, and participants provided frequency of regular soda and sweetened fruit drink consumption [22]. We also measured skin carotenoid status using the Veggie Meter®, which employs pressure-mediated reflection spectroscopy, [23] and is a valid and reliable tool to approximate FV intake through assessing skin carotenoid status [24]. In a prior validation study, the correlation between plasma carotenoids and Veggie Meter® assessed skin carotenoids was 0.71 [24]. Each participant’s finger was scanned three times and the average value of the three measures was used to estimate skin carotenoid status.

### Data analysis

To examine store-level changes, differences between HFSRP and comparison stores in store-level HFS and HEI score changes between 2017 (baseline) to 2020 were analyzed using linear mixed models with an autoregressive error structure. For HFSRP stores only, repeated measure models were used to analyze changes in HFS and HEI from before equipment installation, to 1 to 12 months, 13 to 24 months, and 25+ months after installation.

Customer demographic characteristics were compared by store type (HFSRP vs. comparison) and by year using appropriate statistical tests (ANOVA or chi-square). To examine changes in customer diet behaviors, difference-in-difference models (with and without propensity scores) were used to examine the effect of the HFSRP intervention on the main outcomes of interest: FV servings/day, sugary beverage consumption, and skin carotenoids. Controls were included for age, sex, race (as a proxy for social, environmental, and structural factors), formal education, employment status, annual household income, and shopping frequency at the store where interviewed. We included shopping frequency because shopping frequency could impact the healthfulness of purchases and diet. The store variable was treated as a random effect. Year, and HFSRP status (yes/no), and interaction between these variables were also included to test the effect of the intervention on the main outcomes of interest. Propensity scores were included in the same models to account for differences in some customer demographic characteristics between HFSRP and comparison stores. Propensity scores for each year and HFSRP status combination were estimated using a general logistic regression model with all demographic variables as predictors. Models were stratified by shopping frequency, comparing those who shopped 1–2 times/week or less to those who shopped 3 times per week or more. In a sensitivity analysis, results were examined when using all stores in the program, versus the sub-group of stores (*n* = 6) for which we had at least 3 years of data. All analyses were conducted using SAS version 9.4 (SAS Institutes, Cary, NC).

## Results

### Store characteristics and store-level HFS and HEI changes

The majority of stores were classified (according to the NAICS) as convenience stores, corner stores, small grocery stores (75% of HFSRP stores and 38.5% of comparison stores), or food/gas marts (12.5% of HFSRP stores, 61.5% of comparison stores). The majority of stores accepted SNAP/EBT (87.5% of HFSRP stores, 69.2% of comparison stores), and stores had between 1 and 2 cash registers, with a mean of 1.1 registers in HFSRP and 1.4 registers in comparison stores. Stores had a mean of 4.3 aisles, with HFSRP stores having a mean of 3.1 aisles and comparison stores having a mean of 5.0 aisles.

Bag check data indicate that customers purchased a variety of items, ranging from items for a meal (e.g., corned beef, bananas, biscuits, potato and candy) to cooked meals (e.g., fried fish, cooked green beans, macaroni and cheese, fried chicken gizzards, fried chicken livers, juice drink) to snacks and beverages (e.g., fresh fried peanuts, sodas, water, cheese curls, potato chips).

Table 2 shows store-level HFS and HEI changes over time in HFSRP stores versus comparison stores. Comparing 2017 to 2018, there was a change in the HFS in the expected direction with an increase in HFSRP stores, and a decrease in comparison stores (*p* = 0.052). Similarly comparing 2017 to 2019, there was an increase in HFS in the HFSRP stores and decrease in comparison stores (*p* = 0.031). However, the overall year-HFSRP interaction effect was not statistically significant (*p* = 0.079). Also, there were no differences between HFSRP and comparison store HEI scores generated from the bag check data over time. This indicates that, while the food environment (HFS scores) within the HFSRP stores improved, the customer purchases in the HFSRP stores did not improve when juxtaposed with comparison stores, over time.

**Table 2:** Store-level Healthy Food Supply (HFS) and Healthy Eating Index-2010 of customer purchases (HEI), changes over time, 2017–2020, in Healthy Food Small Retailer Program (HFSRP) Stores versus Comparison Stores

Store-level variables		Years				Overall Effects		
	HFSRP Status	2017	2018	2019	2020	Year	HFSRP	Year x HFSRP
Healthy Food Supply Score	No	5.12 (1.24)	4.51 (1.19)	2.57 (1.01)	3.81 (1.10)	0.415	0.062	0.079
	Yes	4.82 (1.12)	7.70 (1.18)	7.03 (1.09)	5.59 (1.22)			
	p-net*		0.052	0.031	0.363			
Healthy Eating Index-2010 of customer purchases	No	39.5 (3.97)	42.9 (3.90)	37.8 (3.23)	45.3 (3.53)	0.895	0.381	0.277
	Yes	40.1 (3.56)	44.1 (3.87)	45.8 (3.52)	42.2 (3.93)			
	p-net*		0.487	0.732	0.257			

*P-net is the *p*-value for comparing changes from 2017 between HFSRP and comparison stores, so there is no p-net for 2017 because it is the baseline

**Fig. 2:**
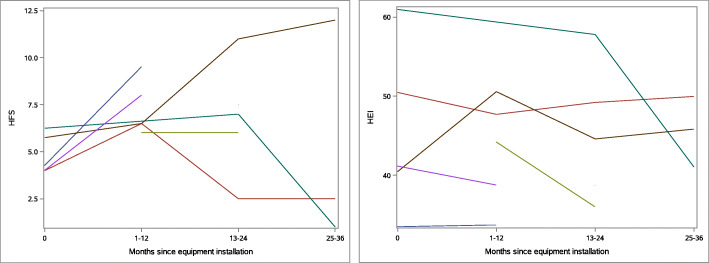
Changes in Healthy Food Supply (HFS) Score (figure on the left) and Healthy Eating Index-2010 of customer purchases (HEI, figure on the right), by months since equipment installation, among Healthy Food Small Retailer Program (HFSRP) stores only

Figure 2 shows changes in store HFS and HEI scores in the months since equipment installation, among HFSRP stores only. There was a borderline statistically significant increase in HFS score among the HFSRP stores comparing the baseline period to that 1–12 months after equipment installation, *p* = 0.055. This borderline difference was only seen within the first year of the program and no differences were seen in year 2 or year 3 of the program. No changes in the HEI were seen over time.

### Individual-level participant characteristics and changes in dietary outcomes

Participant characteristics for HFSRP and comparison store customers in each year are presented in Table 3. Across the study period, there was a mean age range of 41.2–47.3 years, BMI range of 27.7–30.4 kg/m2, mean skin carotenoid scores of 227.3–248.5, and mean FV intake of 3.3–5.1 servings per day. Between 30.3–54.9% of participants were females, and 46.3–82.3% were Black/African American. For several variables, customers were statistically significantly different between HFSRP and comparison stores across and within years, including race, employment status, shopping frequency, mean BMI, FV intake, and sugary beverage intake (See Table 2). For example, in 2019 and 2020, the HFSRP customers were older, on average, than the comparison store customers, and in 2020, there was a statistically significant difference between self-reported FV intake in HFSRP compared with comparison stores (5.10 versus 3.58 servings per day, respectively). In both HFSRP and comparison stores, mean BMI of customers was significantly different over the years; in 2018, on average, HFSRP customers had higher BMI in kilograms/meter squared than comparison store customers. The percent of customers reporting they had previously purchased FV at the store increased significantly in both HFSRP and comparison stores over time (*p* < 0.001) and shopping frequency was significantly different between stores, and across time. These differences could be due to differences in HFSRP versus comparison stores overall.

**Table 3:** Participant characteristics for Healthy Food Small Retailer Program (HFSRP) and comparison store customers (n = 1261) surveyed 2017–2020^a^

	2017		2018		2019		2020		P-value for year effect	
Variable	HFSRP	Comparison	HFSRP	Comparison	HFSRP	Comparison	HFSRP	Comparison	HFSRP	Comparison
n	161	318	109	114	130	184	99	146		
	Mean (SD)	Mean (SD)	Mean (SD)	Mean (SD)	Mean (SD)	Mean (SD)	Mean (SD)	Mean (SD)		
Age (Years)	43.0 (15.1)	43.4 (15.1)	44.9 (13.9)	42.5 (14.8)	**47.3 (15.2)**^b^	**41.2 (15.7)**	**47.2 (15.3)**	**41.5 (15.5)**	0.053	0.377
Body Mass Index, BMI, kg/m^2^	30.4 (6.74)	29.8 (8.37)	**30.1 (6.33)**	**27.7 (6.36)**	29.5 (6.91)	29.6 (7.1)	27.8 (8.08)	28.4 (6.74)	**0.036**	**0.039**
Skin carotenoid score	236.6 (76.5)	232.9 (91.0)	236.8 (81.1)	248.5 (78.0)	243.3 (81.8)	235.4 (72.7)	242.2 (82.5)	227.3 (82.9)	0.868	0.234
2-item Screener of self-reported fruit and vegetable intake, servings/day	3.80 (2.61)	3.96 (3.79)	3.43 (2.29)	3.26 (2.87)	4.06 (3.56)	3.65 (3.41)	**5.10 (5.83)**	**3.58 (2.74)**	**0.008**	0.277
National Cancer Institute Fruit and Vegetable Screener, servings/day	3.61 (3.1)	3.82 (3.19)	3.42 (3.02)	3.53 (3.49)	3.40 (2.91)	3.87 (3.66)	**3.20 (3.41)**	**4.48 (4.71)**	0.787	0.192
Sugary beverages, servings/day	**1.69 (1.94)**	**2.32 (2.49)**	**1.56 (2.02)**	**2.17 (2.3)**	1.55 (2.22)	1.79 (2.32)	1.4 (1.89)	1.76 (2.15)	0.728	**0.033**
	Frequency (%)	Frequency (Percentage)	Frequency (Percentage)	Frequency (Percentage)	Frequency (Percentage)	Frequency (Percentage)	Frequency (Percentage)	Frequency (Percentage)		
Sex (% female)	69 (42.9)	127 (40.3)	33 (30.3)	45 (40.2)	59 (46.8)	100 (54.9)	46 (46.5)	72 (50.7)	**0.042**	**0.005**
Race (%)										
Black	**74 (46.3)**	**235 (74.6)**	**54 (49.5)**	**93 (82.3)**	**64 (51.6)**	**118 (64.8)**	**50 (51.0)**	**87 (62.1)**	0.336	**<.001**
White	**76 (47.5)**	**50 (15.9)**	**48 (44)**	**13 (11.5)**	**46 (37.1)**	**22 (12.1)**	**44 (44.9)**	**21 (15)**		
Other	**10 (6.3)**	**30 (9.5)**	**7 (6.4)**	**7 (6.2)**	**14 (11.3)**	**42 (23.1)**	**4 (4.1)**	**32 (22.9)**		
Income (%)										
< $25,000	**44 (27.3)**	**132 (41.5)**	29 (26.6)	44 (38.6)	52 (40)	86 (46.7)	26 (26.3)	56 (38.4)	0.139	0.479
> = $25,000	**92 (57.1)**	**144 (45.3)**	66 (60.6)	58 (50.9)	61 (46.9)	73 (39.7)	54 (54.5)	66 (45.2)		
Income (missing)	**25 (15.5)**	**42 (13.2)**	14 (12.8)	12 (10.5)	17 (13.1)	25 (13.6)	19 (19.2)	24 (16.4)		
Education (% some college)	**76 (47.2)**	**96 (30.4)**	47 (43.5)	37 (33)	63 (48.5)	76 (41.8)	**60 (60.6)**	**53 (37.1)**	0.078	0.069
Employed (% yes)	**111 (69.4)**	**180 (57.0)**	**79 (74.5)**	**58 (52.3)**	72 (56.3)	106 (57.9)	64 (65.3)	90 (63.4)	**0.021**	0.347
Previously purchased fruits or vegetables at the store (% yes)	36 (22.5)	68 (21.7)	25 (24.5)	22 (19.8)	55 (45.1)	60 (34.5)	45 (48.4)	59 (41.8)	**<.001**	**<.001**
Shopping frequency at the store (%)										
1-2x/week or less	**79 (53.7)**	**81 (25.7)**	**64 (59.3)**	**21 (18.6)**	53 (42.4)	61 (34.9)	**56 (58.3)**	**43 (30.1)**	0.127	**<.001**
3-6x/week	**29 (19.7)**	**71 (22.5)**	**24 (22.2)**	**38 (33.6)**	34 (27.2)	62 (35.4)	**20 (20.8)**	**49 (34.3)**		
1x/day or more	**39 (26.5)**	**163 (51.7)**	**20 (18.5)**	**54 (47.8)**	38 (30.4)	52 (29.7)	**20 (20.8)**	**51 (35.7)**		

^a^*HFSRP* Healthy Food Small Retailer Program Stores

^b^Bold faced numbers mean a statistically significant difference between HFSRP and comparison stores within a year

Table 4 shows changes in customer-level FV intake, sugary beverage intake, and skin carotenoid scores over time. Analysis using difference-in-difference models (with propensity scores), revealed a significant effect of the intervention on the NCI Screener measure of FV (servings/day), in the opposite direction of what was expected, such that FV intake (servings per day) for customers in comparison stores increased more over time than intake of those in HFSRP stores (*p* = 0.050). There were no other statistically significant intervention effects on the outcomes of interest. There was no difference in results when propensity scores were not used, or when using all stores, versus the sub-group of stores (*n* = 6) for which we had at least 3 years of data (data not shown). In models stratified by shopping frequency (Table 3), among those shopping 1–2 times/week or less, there was a significant (*p* = 0.015) interaction between year and HFSRP status on FV intake (servings/day). This result indicates that among HFSRP store customers, there was first an increase, and then a decrease in FV intake, but that this behavior was reverse in comparison stores, such that in comparison stores, first FV intake decreased, then FV intake increased.

**Table 4:** Propensity score adjusted changes in Fruit and Vegetable (FV) intake, skin carotenoids, and sugary beverage intake over time, with propensity scores, and using only customers from the six stores with data for multiple years. These models are adjusted for age, gender, race, education, employment, income, and shopping frequency, except when stratified by shopping frequency

Outcome	HFSRP	Year				Overall effects		
		2017	2018	2019	2020	Year	HFSRP	Year x HFSRP
**AMONG ALL CUSTOMERS (*n*** **= 1222):**		**Means (SE)**				**P (df = 3)**	**P (df = 1)**	**P (df = 3)**
National Cancer Institute FV Screener,^21^ servings/day *N* = 1172	No	3.5 (0.33)	3.3 (0.36)	3.4 (0.35)	4.1 (0.36)	0.958	0.222	0.050
	Yes	3.4 (0.38)	3.2 (0.37)	3.3 (0.36)	2.7 (0.37)			
	p-net		0.875	0.912	0.034			
2-item screener, FV servings/day^20^	No	3.9 (0.29)	3.1 (0.31)	3.4 (0.31)	3.4 (0.31)	0.046	0.341	0.220
*N* = 1185	Yes	3.6 (0.32)	3.2 (0.32)	4.3 (0.31)	3.8 (0.31)			
	p-net		0.444	0.040	0.193			
Veggie Meter® skin carotenoid score	No	228.2 (7.22)	239.7 (7.53)	227.8 (7.37)	218.7 (7.40)	0.159	0.383	0.186
*N* = 1167	Yes	239.6 (7.76)	228.1 (7.87)	242.9 (7.47)	226.8 (7.49)			
	p-net		0.078	0.784	0.794			
Sugary beverage servings/day	No	2.0 (0.22)	2.0 (0.24)	1.7 (0.24)	1.6 (0.24)	0.189	0.498	0.944
*N* = 1203	Yes	1.7 (0.26)	1.8 (0.26)	1.6 (0.25)	1.5 (0.25)			
	p-net		0.838	0.576	0.616			
**AMONG CUSTOMERS WHO SHOPPED IN THE STORE 1–2 TIMES/WEEK OR LESS (*n*** **= 458)**								
National Cancer Institute FV Screener, ^21^ servings/day *N* = 430	No	3.6 (0.50)	2.5 (0.66)	2.9 (0.50)	4.2 (0.53)	0.175	0.995	0.015
	Yes	3.5 (0.45)	4.1 (0.43)	2.7 (0.45)	2.9 (0.44)			
	p-net		0.057	0.978	0.129			
2-item screener, FV servings/day^20^	No	4.2 (0.51)	3. 6 (0.67)	3.3 (0.50)	4.4 (0.49)	0.197	0.180	0.253
*N* = 442	Yes	3.8 (0.41)	3.7 (0.38)	4.7 (0.42)	4.8 (0.39)			
	p-net		0.589	0.048	0.326			
Veggie Meter® skin carotenoid score	No	253.8 (12.7)	245.4 (16.9)	237.4 (12.3)	226.6 (12.5)	0.261	0.619	0.663
*N* = 429	Yes	245.6 (10.8)	225.0 (10.8)	239.9 (11.1)	232.9 (10.6)			
	p-net		0.592	0.610	0.479			
Sugary beverage servings/day	No	1.7 (0.31)	1.7 (0.41)	1.1 (0.30)	1.2 (0.30)	0.805	0.285	0.261
*N* = 444	Yes	1.2 (0.26)	0.9 (0.25)	1.2 (0.27)	1.4 (0.25)			
	p-net		0.731	0.240	0.149			
**AMONG CUSTOMERS WHO SHOPPED IN THE STORE 3–6 TIMES/WEEK OR MORE (*n*** **= 764)**								
National Cancer Institute FV Screener,^21^ servings/day	No	3.5 (0.43)	3.4 (0.45)	3.7 (0.47)	3.9 (0.47)	0.245	0.173	0.322
*N* = 709	Yes	3.2 (0.55)	2.2 (0.58)	3.7 (0.53)	2.7 (0.54)			
	p-net		0.288	0.737	0.266			
2-item screener, FV servings/day^20^	No	3.9 (0.34)	3.0 (0.33)	3.6 (0.36)	3.0 (0.35)	0.046	0.888	0.634
N = 709	Yes	3.3 (0.42)	2.9 (0.44)	3. 9 (0.40)	3.2 (0.40)			
	p-net		0.547	0.221	0.314			
Veggie Meter®, skin carotenoid score	No	217.6 (9.8)	234.2 (10.3)	222.7 (10.8)	213.5 (10.7)	0.097	0.274	0.549
*N* = 706	Yes	238.5 (12.7)	237.4 (13.3)	246.5 (12.2)	219.6 (12.5)			
	p-net		0.316	0.875	0.419			
Sugary beverage servings/day	No	2.2 (0.28)	2.1 (0.30)	1.9 (0.31)	1.8 (0.31)	0.026	0.810	0.286
*N* = 725	Yes	2.2 (0.37)	2.5 (0.38)	1.7 (0.36)	1.3 (0.36)			
	p-net		0.281	0.793	0.397			

## Discussion

To better understand whether healthy corner store initiatives are a effective investment for public health nutrition, a 4-year, observational, natural experiment study of stores participating in the HFSRP was conducted. Findings indicate there were modest improvements in the average healthfulness of foods/beverages available in participating stores, and no improvements in healthfulness of customer purchases. In cross-sectional analysis of individuals, there were changes in self-reported FV intake over time; however, counter to what was expected, customers in comparison stores increased FV intake more than those in HFSRP stores.

The lack of conclusive evidence of improvements in purchases and eating patterns over time could be due to the duration of the HFSRP or the fact that it is very difficult for small food retailers to stock and promote healthier foods and beverages. Stores signed an agreement stating they would stock healthier foods and beverages for 24 months; after this period, store owners could stock this equipment with any items they wished. Following the HFSRP contract period, we found that some of the stores opted to continue stocking the healthier items and others did not. Other policy evaluations, such as that of the Minneapolis Staple Foods Ordinance, [5] have seen limited improvements in stocking. Taken together, results indicate that stocking and promoting healthy foods and beverages within small food retail settings is challenging.

While there were initial improvements in HFS scores, the HFSRP intervention may have been insufficient to produce sustained changes in healthy food and beverage stocking behavior due to a variety of factors including produce distribution, pricing and cost structures, and manager/owner preferences and/or knowledge about healthy foods and beverages. Furthermore, all of the HFSRP stores were independent retailers, and such stores may have a harder time sourcing healthier food and beverage items compared to chain or franchise stores. For example, likely due to their relative resources and infrastructure, chain or franchise stores were found to be more able to change food and beverage items that were stocked compared to independent operators [25]. Indeed, anecdotal evidence suggests that some of the barriers to stocking healthy produce were related to challenges with produce distribution/procurement issues, and also produce spoilage and equipment malfunction. In-depth interviews among HFSRP store owners suggested that some of the HFSRP storeowners had produce spoilage and that may have inhibited their stocking practices [26]. In addition, the owners mentioned that additional technology and technical assistance would have helped with promotion of the healthier items, as well as would have helped meet the tracking and inventory requirements [26]. These barriers were similar to those seen in previous studies. Mayer et al., for example, found that storeowners faced difficulties purchasing high quality produce at an affordable price in small batches [27]. A study in rural eastern NC found storeowners wanted to stock healthy food items, but were skeptical regarding customer demand for them [28]. Future healthy corner store programs and policies should include storeowners in healthy eating interventions to ensure that barriers to stocking and promoting healthy foods are considered and addressed [27].

HFSRP storeowners indicated their customers were interested in learning more about healthy eating but, due to limited HFSRP resources, it was difficult to adequately advertise and promote the healthier options. In some cases, there were partnerships with the local health department that helped to promote new, healthy foods and beverages. Future policies of this type could include language to facilitate public-private partnerships, which would potentially improve the dietary impact of the policy. Additional behavioral economics strategies and store owner trainings were successful in stores in Baltimore, Maryland, and could be implemented in future healthy corner store interventions to increase program impact [29]. Furthermore, it could be that support for stocking healthy foods and beverages in small stores is not effective unless reinforced in the social support systems, schools, and other community venues such as in the intervention study conducted by Trude et al. [30, 31] The relative prices of produce and other healthy options may have been another reason the HFSRP was not more impactful. For future intervention approaches, pricing strategies could potentially improve store stocking of heathy food and beverage items [32]. Increasingly, evidence suggests multi-component interventions, incorporating stakeholders from various community sectors and agencies, would be most effective at changing individual and household eating patterns.

The lack of sustained significant changes in HFS, HEI and customer intake could also be due to inadequate statistical power to detect changes, since fewer stores than anticipated participated in the HFSRP and some of the HFSRP stores closed over time. For example, our power analysis indicated that eight HFSRP and eight comparison stores were needed each year for adequate power; our store sample size was limited due to the number of stores that applied and were selected to be included in the HFSRP. Another factor potentially affecting HFSRP impact on HFS scores is at least one of the comparison stores applied for WIC vendor approval; this would inflate HFS scores in the comparison store group since the HFS measure is based upon WIC-approved foods. A further limiting factor is that a single intervention such as the HFSRP, that relies on stocking more health foods and beverages without any other supports for merchants and incentives for consumers will not to lead to significant changes in purchasing or diet.

This study provides a rigorous evaluation of a state-level healthy corner store policy initiative including rural, underserved areas, whereas prior studies have been largely focused in urban settings [3]. Among this study’s strengths, our natural experiment included data from a large and varied sample of stores and residents, and we also used objective measures of purchases and fruit and vegetable intake among customers. While the current study employed objectively assessed fruit and vegetable intake (Veggie Meter® scores) and bag checks to assess purchasing behavior, future studies could use additional sales tracking methods, as done by Sadeghzadeh et al., [33] wherein stickers were placed on food items and store owners/staff removed the sticker and placed it on a tracking sheet when the item was purchased.

This study also used a convenience sample of customers in each store and was unable to follow the same customers over time. More longitudinal analyses are needed to better understand the long-term impacts of these types of interventions. Other study limitations include the fact that customers differed in their demographic characteristics and their behavior, both across store types (HFSRP versus comparison) and across years, and that assessments were conducted among store customers and not necessarily the consumers of the healthy foods and beverages purchased at the stores. A further limitation is that a majority of customers interviewed shopped at a small store 3–4 times per week, indicating limited generalizability—however, the stores in the study were in food deserts, and thus, may have been one of the only options available to local residents with limited transportation. Examining behaviors of food desert residents is a strength of our study.

The sample of HFSRP stores was different than the sample of comparison stores, despite our attempts to match the HFSRP and comparison group stores. Such differences may have resulted in differences in the customer samples (e.g., the difference between self-reported FV intake in HFSRP compared with comparison stores in 2020, the differences in store-level HEI at baseline). However, we used propensity score matching to reduce the effects of these differences in our analyses. In addition, we did not match stores on WIC status, which could have made it more difficult to determine the true effects of the HFSRP on stock and customer behaviors. Furthermore, we did not collect data on survey acceptance rate so we cannot compare responders to non-responders. Also, while many individuals surveyed reported that they shopped quite frequently at the stores where surveyed, they were most likely not using the small stores as their main shopping venue, as prior research indicates that most Americans grocery shop at larger supermarkets and big box stores [34]; thus, small store interventions may influence a relatively small part of an overall eating pattern, unless shopping behaviors change or the customer is a food desert resident with limited transportation and is thus more reliant on the food store. Finally, we were forced to stop data collection in 2020 due to Covid-19 and this limited our sample size in 2020.

## Conclusions

While the HFSRP offers a strong step towards provision of healthier foods and beverages in underserved areas, this program did not address a variety of factors such as customer education, pricing incentives, advertising/placement, and distribution of healthier foods and beverages to small stores. In future iterations of the HFSRP or other similar initiatives, more attention should be given to the broader contextual issues and evidence-based approaches to promote the consumption of healthier foods and beverages, including additional technical support for retailers and incentives for consumers. As one example, future initiatives should better integrate these environmental supports with innovative nutrition education and promotion programs and, particularly if the stores are required to accept both SNAP and WIC, perhaps include complementary nutrition education and promotion programs such as SNAP-Education and WIC nutrition education. Along with nutrition education and social marketing, SNAP-Ed can now also use policy, systems, and environmental (PSE) changes to deliver nutrition messages and has been utilized to supporting healthy retail food outlet interventions [35, 36]. Furthermore, the HFSRP had limited funding for personnel to implement and evaluate the program; it would be beneficial for future program appropriations to include additional funding for such staffing needs. As others have suggested, [1, 5] determining financially profitable models and supply chain logistics for small stores to stock and promote healthier foods and beverages is critical to the long-term success of such initiatives. Altogether, more comprehensive, statewide solutions that complement existing initiatives are needed across food retail but also within other community settings including schools and worksites, in order to shift demand and ultimately, health outcomes.

## Data Availability

The datasets used and/or analyzed during the current study are available from the corresponding author on reasonable request.

## Abbreviations

HFS: Healthy Food Supply
HEI: Healthy Eating Index
NC: North Carolina
FV: Fruit and Vegetable
HFSRP: Healthy Food Small Retailer Program
SNAP-Ed: Supplemental Nutrition Assistance Program-Education
WIC: Special Supplemental Nutrition Program for Women, Infants and Children
NCI: National Cancer Institute

## Declarations

### Recruitment information

Customers recruited for the study were approached as they were finishing up their purchases and in some cases, were recruited by store owners, and referred to the research assistants to complete the bag check and intercept interview. We used all available data in analyses, and have shown how sample sizes differed by analyses, in Table 4. Table 3 shows the differences in demographic factors over the years, and propensity score analyses were used to adjust for these differences across stores and over the different years of data collection.
